# Recruitment and retention of staff in rural dispensing primary care practice: a qualitative inquiry

**DOI:** 10.3399/BJGPO.2023.0130

**Published:** 2024-02-21

**Authors:** Rosina Cross, Sinead TJ McDonagh, Emma Cockcroft, Malcolm Turner, Matthew Isom, Robert Lambourn, John L Campbell, Christopher E Clark

**Affiliations:** 1 Exeter Collaboration for Academic Primary Care, Department of Health and Community Sciences, Faculty of Health and Life Sciences, University of Exeter, Exeter, UK; 2 Dispensing Doctors’ Association Limited, North Yorkshire, UK; 3 Cheviot Primary Care Centre, Wooler, Northumberland, UK

**Keywords:** Primary health care, rural health services, dispensing, pharmacy, workforce, qualitative research

## Abstract

**Background:**

Rural primary care practices struggle to employ and retain staff, and existing literature regarding recruitment and retention is focused on doctors. Shortages of qualified staff affect practice functioning, quality of care, and patient experience. Dispensing of medications is a rural service valued by patients. However, little is known about how dispensing services are valued by practices or related to the recruitment and retention of staff.

**Aim:**

To understand barriers to, and facilitators of, joining and remaining in rural dispensing practice employment, and to explore how rural practices value dispensing services.

**Design & setting:**

Qualitative inquiry in rural primary care practices across England.

**Method:**

Semi-structured interviews with rural dispensing staff were undertaken, audio-recorded, transcribed verbatim, and analysed using framework analysis.

**Results:**

In total, 17 staff from 12 practices across England were interviewed between June and November 2021. Reasons for taking up employment in rural dispensing practices included perceived career autonomy, development opportunities, and preference for working and living in a rural setting. Skills required for dispensers’ roles balanced against low wages were a barrier to recruitment. For nurses, barriers included perceived lack of knowledge around their role in rural care. Revenue from dispensing, opportunities for staff development, job satisfaction, and positive work environments drove retention of staff. However, negative perceptions of rural practice, travel difficulties, lack of applicants, and insufficient remuneration for roles were barriers to retention.

**Conclusion:**

Barriers to, and facilitators of, rural primary care recruitment and retention vary by role, and include factors unique to the rural setting.

## How this fits in

Recruitment and retention of multidisciplinary primary care staff to rural dispensing practice is challenging; little is known about the barriers and facilitators to working and remaining in employment in this setting. Dispensing medicines is a unique aspect of rural primary care and contributes to financial stability of practices, yet not much is known about its relevance to staff employment. Low wages relative to skillset in dispensing roles, and poor knowledge surrounding rural nursing roles, present challenges for recruitment, while career autonomy, job satisfaction, and work environment are key drivers of recruitment. Both revenue and career development opportunities from dispensing, job satisfaction, and work environment are the main drivers of staff retention, while negative perceptions of rural primary care, perceived travel difficulties, lack of skilled applicants, and difficulties balancing dispensing skills with the wages offered to dispensers are key challenges to staff retention.

## Introduction

Most patients in England enjoy easier access to a community pharmacy than to general practice.^1^ In rural areas this is not the case; rural practices can hold a right to dispense prescription medications to their patients. In general, such dispensing rights apply where patients live >1 mile (>1.6 km) from the nearest registered pharmacy. In 2022, there were >1100 such rural dispensing practices in England, approximately 13% of all primary care practices, supplying medication to around 3.5 million NHS patients.^2^ Dispensing of medications is a service valued by patients and significantly contributes to financial stability of rural dispensing practices.^2^ Dispensing may also contribute to improved medication adherence.^3^

The UK National Centre for Rural Health and Care (NCRHC) recently highlighted the higher costs of delivering primary health care in rural compared with urban settings. Its report identified unique factors affecting rural recruitment, including migration of younger people from rural areas, high local employment rates, and absence of rural proofing in workforce planning.^4,5^ In Scotland, rural GPs report higher rates of intention to leave primary care employment in the next 5 years than their non-rural counterparts.^6^ However, primary care relies on multidisciplinary teams, therefore, an understanding of factors affecting recruitment and retention for all rural primary care team members is desirable to address current and future workforce challenges.

Workforce studies usually focus on GPs.^7–9^ Comparatively little is known about workforce challenges for other multidisciplinary primary care team members in general; this is particularly true for rural practices where the additional workload implications and additional recruitment, training, and retention challenges for GPs, pharmacists, and dispensers in dispensing practices are poorly described.

Rurality poses unique challenges to recruitment and retention of staff; for example, the role of place and environment assumes an importance not recognised in urban settings.^10^ Inequalities in access to health care for rural primary care staff members is another facet of this.^11^ Dispensing staff often live in close proximity to, and are frequently registered as patients with, their practice, lacking easily accessible primary healthcare alternatives for themselves and their families.^12^ Proximity issues can arise for staff and their families, including perceived lack of anonymity in accessing health care, concerns over confidentiality of medical records, and conflicts of employer and doctor roles for staff patients not fit to work.^13^ The General Medical Council (GMC) cautions doctors against providing medical care *‘to anyone with whom you have a close personal relationship’*, while the main English medical defence organisations recognise the dilemma of having staff as patients, advising either against having current staff as practice patients or separating employee and patient roles clearly.^14–16^ Rural practice staff may, therefore, be restricted in their choice of GP. It is unknown how such factors impact on the recruitment and retention of non-GP primary care staff in rural settings.

The primary aims of this study were to understand the barriers to, and facilitators of, taking up and/or remaining in rural dispensing primary care employment, and to elucidate the value placed on dispensing from the perspectives of the multidisciplinary primary care team. A secondary aim was to gain an understanding of potential conflicts in treating staff as patients, and how these might impact recruitment and/or retention.

## Method

### Research design and sampling

Remote (online) semi-structured interviews were conducted with multidisciplinary primary care team members across England. Participants were recruited via Clinical Research Networks and snowballing. Study adverts were also circulated on social media (see Supplementary Information S1a for recruitment materials). A purposive sampling strategy was used to maximise sample diversity across primary care staff roles. Full inclusion and exclusion criteria are listed in Table 1.

**Table 1.** Participant eligibility

Inclusion criteria	Multidisciplinary primary care staff members Aged ≥18 years Individuals currently employed or previously in full- or part-time permanent positions (including those in salaried or partner positions) Speak fluently in English language
Exclusion criteria	Individuals aged <18 years Individuals in non-permanent or temporary roles, including locum, trainee, junior, or community or district clinical staff

### Data collection procedures and informed consent

A semi-structured topic guide was co-developed by the study management group and patient and public involvement (PPI) adviser before piloting with local primary care staff (See Supplementary Information S1b). Once written informed consent was received (see Supplementary Information S1c), individual interviews were conducted with participants using video-conferencing software (Zoom or Microsoft Teams, depending on participant preference). Interviews were video- and audio-recorded, and audio was transcribed by an external company under a confidentiality agreement.

### Analysis

Interview data were analysed using framework analysis,^17^ facilitated by NVivo software (version 12). A predetermined framework with four themes (drivers of recruitment, challenges to recruitment, drivers of retention, and challenges of retention) was employed to meet the aims of the study. Five research team members (RC, SM, CEC, EC, and MT) analysed the data, first familiarising themselves with the data by reading interview transcripts before starting coding. At least two research team members, including a PPI adviser, coded data independently for each interview and iteratively reviewed the framework before discussing themes with the wider research team.

The lead researcher (RC) paraphrased participant responses during interviews to ensure correct interpretations, increase credibility, and minimise bias.^18,19^ Rigour was enhanced by the following: a) researcher reflection on interview field notes, themes, and coding; and b) the use of multiple multidisciplinary coders. Transparency was increased by creating a detailed audit trail, and extensive critical discussion of themes between coders.^18,19^

## Results

Semi-structured interviews were conducted with 17 participants between June and November 2021 (Table 2). Mean interview time was 35 minutes (range 18–78 minutes).

**Table 2.** Participant characteristics

Participant number	Time at general practice(at time of the interview), years	Role at general practice	Estimated patient population size of practice, *n*
P001	9	GP and partner	5000
P002	28	Full-time GP	4270
P003	6	Practice manager	4400
P004	5.5	Practice manager	11 000
P005	17	Practice nurse	5000
P006	8	Senior partner and GP	17 300
P007	2	Clinical coder and summariser	26 000
P008	7.5	Partner and GP trainer	2300
P009	6	Healthcare assistant	5000
P010	5	Student nurse and dispenser	5500
P011	16	Practice business manager	16 500
P012	21	Practice manager	4000
P013	4.5	Social prescriber	17 000
P014	14	Nurse prescriber	10 700
P015	0.5	Business administrator	20 000
P016	7	Dispensary manager	4000
P017	10	Lead dispenser	9000
Mean (range)	9.8 (0.5–28)		9822 (2300–26 000)

### Findings

**Figure 1.**
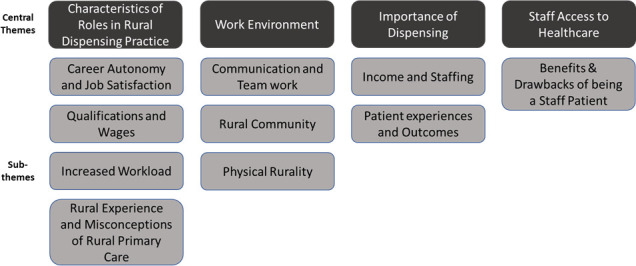
Hierarchy of themes

The following four central themes relating to drivers of and challenges to recruitment and retention of staff were identified: characteristics of roles in rural dispensing practice; work environment; importance of dispensing; and staff access to health care. Central themes and their sub-themes are illustrated in Figure 1.

#### Characteristics of roles in rural dispensing practice

This theme was identified as influencing both recruitment and retention of staff, with sub-themes discussed below.

##### Career autonomy and job satisfaction

Career autonomy, driven by practice needs and personal desire, was perceived as influencing recruitment and retention. Being part of a smaller team, staff needed and relished opportunities to diversify skills and adopt numerous roles. This brought a sense of autonomy and job satisfaction:

*‘… It’s lovely having the autonomy to pursue my interests and do as I want to do — my partnership’s very flexible like that … ’* (Participant [P]001, GP partner, Devon)

Despite facing challenges, such as staffing and pressure from the COVID-19 pandemic, many clinical and non-clinical staff believed their roles were stable and offered good pensions.

##### Qualifications and wages

Clinical staff described their roles as being relatively well paid:

*‘... there are some negatives, but they are — by far and away — outweighed by the positives of the job’s stability, income.’* (P002, GP, Oxfordshire)

However, this was not the case for administrative and other primary care team members, such as dispensers, whose wages were significantly lower relative to the skill set and responsibility required by the role:

*‘… we’re classed as health care so people are thinking “I don’t want to have all that pressure on me, I could go for example, work in* [a local supermarket] *on the checkouts for the same money and there isn’t the pressure and responsibility".’* (P017, lead dispenser, North Yorkshire)

The issue of low wages was perceived as a deterrent for prospective candidates, with many practices struggling to recruit dispensers:

*‘… we’ve been looking to recruit and we’ve had to readvertise three times, and we’ve not had an applicant so … ’* (P017, lead dispenser, North Yorkshire)

##### Increased workload

Increased workload was a characteristic of all roles interviewed, driven by increased practice size, staff shortages, and pressures driven by the COVID-19 pandemic. Increased workload sometimes drove recruitment, with new roles advertised to accommodate the increase. While none of the staff interviewed highlighted increased workload as a reason they would consider leaving, many suggested it could have influenced decisions to leave made by former members of staff.

##### Rural experience and misconceptions of rural primary care

Lack of rural experience and knowledge about rural primary care were identified as challenges to recruitment for some roles, particularly for nurses and non-clinical staff (for example, dispensers), with many posts taking months to fill. Nurses suggested that this was owing to misconceptions around the nursing role in rural primary care. If nurses had not previously trained in a rural setting, they were less likely to apply for rural posts once qualified. Misconceptions about rural practice and lack of knowledge of the dispensing role were also perceived reasons for difficulties in recruiting dispensers:

*‘So, when you advertise — particularly in our communities — dispensers will often work in big towns … the job they’re asked to do with us … is very different from the job they’re doing as a dispenser under direct supervision from a pharmacist. So, they don’t quite understand the differences and, therefore, they’re put off … ’* (P001, GP partner, Devon)

### Work environment

Work environment was identified as a key contributor to work–life balance in rural dispensing practice, typically influenced by personal, social, and spatial factors, as illustrated in the sub-themes below.

#### Communication and teamwork

Communication and teamwork were drivers of recruitment and retention of staff. Participants stated that the positive team environments encountered during training was a key motivator for joining a rural dispensing practice, once qualified. A well-functioning team was described as staff feeling supported within their role, having the opportunity to seek support or advice, and having clear lines of communication across the multidisciplinary team:

*‘... we’re a very close-knit strong team … we’re good friends as well as colleagues and we have a very good relationship with one another.’* (P014, nurse prescriber, North Yorkshire)

A sense of obligation to the team and appreciation of the positive work environment motivated staff to remain in post.

#### Rural community

Participants valued the rural community where they lived:

*‘… so we migrated up here, so I think the attraction of the scenery, the active life up here, was the big draw.’* (P006, senior partner and GP, Cumbria)

Rural scenery, sense of community, and an environment that supported active living was thought to contribute to achievement of a work–life balance:

*‘… it felt important to us to raise the kids somewhere like this … There’s a very strong community here.’* (P003, practice manager, Cumbria)

Alternatively, several doctors preferred to live out-of-area to remain independent of the community; this was not perceived as impacting recruitment and retention of doctors, with many simply living in nearby communities:

*‘… it’s my choice to live this far away … the days have gone where GPs live on sites, and we had to provide that urgent care for out of hours … my generation are very keen we don’t live in our areas, so that we can still be our own people, although my patients often ask when I’m moving in!’* (P001, GP partner, Devon)

#### Physical rurality

The physical rurality of practices was both a driver and a challenge to recruitment and retention. Positive aspects were reported:

*‘It’s just quite relaxing sometimes … in the springtime there would just be lambs bouncing around right outside your window.’* (P010, student nurse and dispenser, Northumberland)

The positive aspects were cited as reasons to remain:

*‘… largely nice patients, largely a great working environment — eight minutes journey to work.’* (P002, GP partner, Oxfordshire)

However, rurality was perceived as a challenge to recruitment and retention:

*‘I was also slightly mindful of staff and recruitment … it’s often this mindset of, “There’s a lot of lanes here — it’s a long way to get here.” So, it’s normally those sort of things that are more challenging for us with staff recruitment.’* (P001, GP partner, Devon)

The concern of recruitment and retention was compounded by poor public transport, difficult commutes in the winter, and alternative job opportunities in urban areas where many currently lived. Travel difficulties combined with lower relative wages in some roles (administrative staff, dispensers, and healthcare assistants) presented a further challenge to recruitment and retention.

### Importance of dispensing

Dispensing played a complex role in recruitment and retention of staff, acting as both a driver and a challenge; illustrated in the sub-themes below.

#### Income and staffing

GP partners reported that dispensing allowed them to pay higher wages than non-dispensing practices, which helped them attract applicants to roles:

*‘The dispensary — it does afford us the ability to pay above the average, which — in a way — we have to do anyway because of where we live, so we do pay our staff more for working for us.’* (P002, GP partner, Oxfordshire)

GP partners and practice or business managers also identified dispensing as key to a well-funded, well-run practice, without which overall patient experience would suffer:

*‘... it’s really important both for our community because of how rural we are — it’s important for people — and also, it’s really important for us — financially.’* (P003, practice manager, Cumbria)

An unexpected driver of recruitment was that a dispensary provided development opportunities for non-clinical staff to upskill; for example, by training as dispensers. This was a motivator for them joining and remaining in the practice. However, there was a complexity attached to this: further training under a pharmacist (to progress to National Vocational Qualification [NVQ] level 3 [pharmacy technician] from NVQ2 [dispenser]) is not feasible in many rural dispensing practices, owing to there being no pharmacists on site. This was said to affect retention; dispensers seeking further training and progression leave rural practice to join other practices or pharmacies:

*‘Level three you do have to do under the supervision of the pharmacist … I’d have probably stuck with the Level 2 … because I wouldn’t have wanted to leave the surgery. But I was really interested in doing my Level 3* [training]*. So … having to maybe do some part-time work at a pharmacy.’* (P017, lead dispenser, North Yorkshire)

#### Patient experiences and outcomes

Participants believed that their ability to provide dispensing services improved both patient experiences and outcomes. Dispensing services were credited for increased access to services, better continuity of care, and greater medication adherence:

*‘… you know that they* [the patient] *can go out of your room and go and collect it* [the medication]*, then they’re more likely to take it than go away with a script … I think they’re more likely to be compliant.’* (P005, practice nurse, Devon)

Without dispensing, patient experiences would be impacted negatively through increased travel time and the costs incurred in accessing medications. For many staff, offering a good service to patients was important for job satisfaction and a motivator to stay in practice.

### Staff access to health care

Staff identified both benefits and difficulties to accessing primary care themselves.

#### Benefits and drawbacks of being a staff patient

GMC guidelines for doctors as patients were recognised; however, for other staff, there was less clarity, and many non-medical staff described receiving care within their practice as either registered or temporary patients. They valued the convenience of this arrangement, considering that travel, work, and family commitments would make it harder to access care elsewhere. Potential issues with being a patient within the workplace were also raised. These included the sensitivity that comes with some diagnoses or illnesses, employer and doctor conflicts of interest, maintenance of confidentiality (both personal confidentiality and that of colleagues), and consequences for work relationships if there are breaches in confidentiality. Despite this, all non-medical staff interviewed remained confident in their healthcare team to provide quality care for them if they were in need:

*‘To my mind, it comes down to a) the doctors, and b) the staff’s integrity. When the doctor’s seeing a staff member, they need to absolutely forget that they’re a staff member and deal with them as a patient … ’* (P003, practice manager, Cumbria)

Many participants reported that staff had refused to be seen elsewhere for treatment, so they felt obliged to continue caring for them within the practice. Some rural practices adopt workarounds to facilitate access to care:

*‘… so, we have an agreement amongst all the local practices that they would register other staff from neighbouring practices who felt that they would prefer to be registered not with their own practice.’* (P008, GP partner, Northumberland)

## Discussion

### Summary

This study explored the barriers to, and facilitators of, recruitment and retention of multidisciplinary healthcare team members in rural dispensing practices in England. A range of factors specific to rural settings and to the ability to dispense medications were identified. Interconnected themes were categorised as characteristics of the role, work environment, importance of dispensing, and staff access to health care.

### Strengths and limitations

While recruitment and retention of doctors has been well researched, findings for other team roles are often missing.^20^ This study aimed to include perspectives on recruitment and retention from an array of primary care team members. The purposive sampling strategy maximised diversity of interviewees, providing rich data based on real-world experiences of rural primary care staff. However, it is acknowledged that the inclusion of additional dispensing staff could have been even more valuable in understanding the mechanisms impacting recruitment and retention of staff working in this role in rural dispensing practice. Thematic saturation was reached during analysis; however, we cannot be sure that the addition of data from other regions of the UK, or from participants who were unavailable for interview, would not have impacted the findings. We specifically sought, but failed to recruit, former primary healthcare professionals who may have added depth to our understanding of retention in this context. Consequently, we are cautious of generalising findings to other populations and primary healthcare systems outside of England. In order to preserve interviewee anonymity, we were unable to give greater geographical or practice details in our report.

### Comparison with existing literature

Recruitment and retention studies usually focus on GPs and generally lack relevance to rural primary care in England.^8,9^ A 2015 Cochrane review found only one relevant study, concluding that further studies are needed to establish the true effects of health service policies on recruitment and retention in rural and other underserved settings.^7^ Recent reports from the NCRHC have acknowledged the higher costs of healthcare delivery in rural compared with urban settings,^5^ and identified migration of younger or working-age people away from rural areas, relatively high rural employment rates, and the absence of a rural component to workforce planning as just some of the unique challenges to rural recruitment.^4^

Teamworking offers several benefits across primary healthcare settings, including improved service provision, reduced patient mortality, enhanced patient satisfaction, and improved staff motivation and wellbeing.^21,22^ The importance of teams and wider support networks is commonly cited in the literature as being a key determinant of recruitment and retention in rural practice and is in line with the findings in this study.^23^

The value placed on rural life as a means of achieving a balance between work and family life is a sentiment echoed in recent work. The importance of family and spousal factors in decisions to join a rural practice and family experiences of rurality and preferences of rural living were pertinent.^24–28^ Many healthcare staff are embedded within their local communities, although for doctors this was not a universal aspect and views differed.

The importance of ‘place’ is emerging as a specific driver to rural recruitment or retention.^10,23^ The complex interdependency of individuals, teams, and local communities affecting not just recruitment and retention of staff in rural dispensing practice but also the health outcomes of patients within these communities reflects the ‘whole-of-society approach’, championed by the World Health Organization. It recognises and values the diverse social foundations, activities, and systems responsible for determining people’s access to resources and opportunities, and the impact this has on health outcomes in wider society.^29^

Rural travel can be both pleasurable and, at times, challenging. Access is compounded by rising fuel and cost-of-living expenses, and diminishing public transport. For these reasons practice staff tended to live locally, bringing valuable local knowledge to the healthcare team. However, it also means that recruitment is often limited to small local pools of potential employees.^30^

Income from dispensing can represent up to half of total income for a fully dispensing practice.^2^ This is vital for funding adequate (but still often, compared with pharmacies, low) wages to attract and retain dispensers and to maintain practice services. It is unusual to recruit a fully trained dispenser, with in-house training being commonplace and valued by staff. However, training beyond NVQ level 2 requires the direct supervision of a pharmacist, which can threaten staff retention as they are often obliged to seek this further training opportunity outside of the rural practice (that is, in a pharmacy).^31^ Dispensing helps to overcome geographical barriers to accessing medications and may contribute to improved medication adherence and patient outcomes;^3,32^ this service is valued by rural patients.^31,33^

GMC guidance on good medical practice requires GPs and their families to be registered outside of their practice.^15^ The guidance is less clear for employed (non-GP) staff, but the GMC states that: *‘Wherever possible, you should avoid providing medical care to anyone with whom you have a close personal relationship.’* However, rural and remote patients (including local staff) are less able to exercise choice of providers in accessing primary care.^11^ Anonymity for staff and their families is also a consideration; rural teenagers have perceived barriers in accessing sexual health services in confidence within their communities and local practices.^13^ Extra-practice services are generally preferred by teenagers for greater anonymity, and schemes, such as pharmacy supply of emergency contraception, can improve overall uptake^34^ yet tend to be located in towns, thereby presenting geographical barriers to access.^35^ Practice proximity to home or school is valued, but balanced with anxiety about visibility of use.^36^ Similarly, uptake of primary pharmacy or accident and emergency services is correlated with convenience and locality, and owing to distance decay is relatively less accessible for rural practice staff and their families.^32^ This study elicited a range of views on staff as patients and identified practical solutions that can be applied to rural proof their provision of primary care.

### Implications for research and practice

Recognition of the different and specific challenges experienced in recruiting to rural or urban practices is needed to promote successful recruitment to, and retention of, staff across all primary care teams. Job satisfaction is complex and components vary. A single contract seems unlikely to meet all needs and a range of solutions to workforce challenges, with appropriate rural proofing of initiatives, are needed to address the current situation.^37^

The importance of prior rural primary care exposure for recruitment has been recognised and was clearly reiterated in this study. The recommendations of the All Party Parliamentary Group for Rural Health and Care to increase, or even mandate, rural exposure in training posts should be taken seriously by educators and policymakers.^38^ Commissioners need to engage with key stakeholders in research exploring how rural dispensing practices can be supported to serve their communities, and the lessons learnt should then guide practice policy change.

Encouragement of pharmacists into practices, driven by primary care practice networks, may offer the potential to overcome the ‘barrier’ for training dispensers beyond NVQ level 2. Integration of pharmacists into primary care is good for patients; if their supervisory and educational roles are fully realised, they may also substantially strengthen rural dispensing teams and practices too.^39^

Defence organisations caution against retaining staff on practice lists; however, there is no practical alternative for many without disruption, such as additional travel time and costs, creating barriers to their personal access to health care.^14,16^ Removal from a practice list owing to employment might also be perceived as punitive or discriminatory. Employed rural staff do, therefore, often remain registered at their place of work, particularly in island and remote settings. This raises potential conflicts and concerns, as previously described. Neither the GMC nor medical defence organisations make particular provision for rural and remote settings; consequently, doctors develop and agree local guidelines with their staff, such as reciprocal arrangements with neighbouring practices where possible, in order to maintain their access to care. Such arrangements are evidently valued and trusted by staff; however, doctors may find themselves consciously or unconsciously in breach of national guidance in offering these services. This is an area where flexibility, local consultation with stakeholders, and rural proofing of blanket national guidance is required in order to avoid discriminatory healthcare arrangements for rural staff.^24,40^

In conclusion, this study has identified important barriers to, and some facilitators of, rural dispensing practice recruitment and retention. Consideration of these findings should enable better understanding of rural dispensing primary care staffing concerns, and emphasises the need for sufficient, future resourcing for healthcare provision in this setting.
